# Digital breast tomosynthesis system concept addressing the needs in breast cancer screening and diagnosis

**DOI:** 10.1117/1.JMI.12.S1.S13010

**Published:** 2024-12-17

**Authors:** Marcus Radicke, Marcel Beister, Stephan Dwars, Joerg Freudenberger, Pilar B. Garcia-Allende, Bernhard Geiger, Katrin Hall, WenMan He, Axel Hebecker, Carina Heimann, Daan Hellingman, Magdalena Herbst, Mathias Hoernig, Thomas Klinnert, Ferdinand Lueck, Ralf Nanke, Ludwig Ritschl, Stefan Schaffert, Sabine Schneider, Daniel Stein, Julia Wicklein, Steffen Kappler

**Affiliations:** a Siemens Healthineers AG, Forchheim, Germany; b Siemens Medical Solutions USA Inc., Malvern, Pennsylvania, United States; c Siemens Healthineers Innovation GmbH & Co. KG, Röttenbach, Germany

**Keywords:** digital mammography, digital breast tomosynthesis, synthetic mammograms, flying focal spot, fast acquisition, high-resolution, wide-angle digital breast tomosynthesis

## Abstract

**Purpose:**

Digital breast tomosynthesis (DBT) has been introduced more than a decade ago. Studies have shown higher breast cancer detection rates and lower recall rates, and it has become an established imaging method in diagnostic settings. However, full-field digital mammography (FFDM) remains the most common imaging modality for screening in many countries, as it delivers high-resolution planar images of the breast. To combine the advantages of DBT with the faster acquisition and the unique in-plane resolution capabilities known from FFDM, a system concept was developed for application in screening and diagnosis.

**Approach:**

The concept comprises an X-ray tube with adaptive focal spot position based on the flying focal spot (FFS) technology and optimized X-ray spectra. This is combined with innovative algorithmic concepts for tomosynthesis reconstruction and synthetic mammograms (SMs).

**Results:**

An X-ray tube with FFS was incorporated into a DBT system that performs 50-deg wide tomosynthesis scans with 25 projections in 4.85 s. Laboratory evaluations demonstrated significant improvements in the effective modular transfer function (eMTF). The improved eMTF as well as the effectiveness of the algorithmic concepts is shown in images from a clinical evaluation study.

**Conclusions:**

The DBT system concept enables high spatial resolution at short acquisition times. This leads to improved microcalcification visibility, reduced risk of motion artifacts, and shorter breast compression times. It shifts the in-plane resolution of DBT into the high-resolution range of FFDM. The presented technology leap might be a key contributor to facilitating the paradigm shift of replacing FFDM with DBT plus SM.

## 1 Introduction

Full-field digital mammography (FFDM) and digital breast tomosynthesis (DBT) are recommended in breast cancer screening and diagnosis.^1^^,^^2^ As of today, DBT is rapidly emerging as the new standard of care for breast cancer screening in the United States, and FFDM is still the most used method in countries outside the United States. Screening studies demonstrated higher cancer detection rates and reduced recall rates with DBT compared with FFDM; however, effects are dependent on the screening setting, with greater improvement in cancer detection rates in European studies (biennial screening) and a reduction in recall rates in U.S. studies (with high baseline recall).^3^

In comparison with FFDM, some of the main needs in breast cancer screening are better addressed by the DBT technology. Acquiring data at different angles results in additional tomographic information. DBT systems with a wider angular range have a higher out-of-plane spatial resolution and consequently have a better ability to resolve low-contrast soft tissue findings, i.e., architectural distortions and masses, from overlapping tissue. Narrow-angle DBT systems can be designed to have shorter acquisition times and, in combination with higher kVp settings and less projection images, provide, until now, slightly easier perceptibility of calcifications.^4^^–^^7^ Importantly, most screen-detected cancers are associated with soft tissue findings, whereas less than 20% are associated with calcifications only.^8^

As DBT is a pseudo-three-dimensional imaging technique, reduced compression is possible due to better separation of the tissue structures in the out-of-plane direction. The Malmö Breast Tomosynthesis Screening Trial demonstrated a 34% higher cancer detection rate using wide-angle DBT, whereas compression force was 40% lower compared with FFDM.^9^ One of the main reasons that refrain women from taking part in screening programs is the pain caused by compressing the breast.^10^ Importantly, low attendance rates limit the effect of breast cancer screening programs with respect to the aim of reducing mortality.^11^

Finally, the synthetic mammogram (SM) was developed to provide FFDM-like images computed from DBT datasets without additional radiation dose. It is considered faster and easier to compare a time series of two-dimensional (2D) images than to scroll through a time series of DBT slices. In particular, 2D images provide an excellent overview and facilitate the detection of microcalcification clusters and right/left asymmetries. Studies have shown that DBT plus SM results overall in superior diagnostic accuracy compared with FFDM alone^12^^,^^13^ and non-inferior diagnostic accuracy compared with DBT plus FFDM,^12^^,^^14^ respectively. Nevertheless, in current system concepts, some malignant microcalcifications can be less conspicuous on DBT and SM compared with FFDM.^15^

A novel system concept, recently commercialized and Food and Drug Administration–approved under the name MAMMOMAT B.brilliant (Siemens Healthcare GmbH, Erlangen, Germany), was developed to facilitate the paradigm shift of replacing FFDM by DBT plus SM in breast cancer screening. This next-generation 50-deg wide-angle DBT system retains all the benefits of wide-angle DBT systems and realizes short acquisition times known from narrow-angle systems. As will be shown in this study, it shifts the in-plane resolution of DBT even into the high-resolution range of FFDM.

## 2 Purpose and Methods

### 2.1 Optimization of the Spectrum and Its Dependency on the DBT Angle

During the past decades, and especially since the introduction of digital detectors, there have been significant efforts in improving efficiency in the field of mammographic X-ray imaging.^16^^–^^20^ The optimization has been primarily related to the notion of achieved image quality in relation to applied patient dose via tailoring of the X-ray spectrum.^19^^,^^20^ With the advent of tomosynthesis and the overall awareness of potential patient movement artifacts being linked to the exposure time, tube power limitations should be added to the optimization’s problem statement.

In our work, we addressed image quality in terms of the well-established definition of the object-related contrast-to-noise ratio (CNR) and weighted it against the costs of average glandular dose (AGD) and electrical energy consumption. A simplified simulation approach (one-dimensional model and considering quantum noise as the single noise source) for imaging of aluminum sheets and 1-mm calcium over poly(methyl methacrylate) (PMMA) and breast tissue backgrounds in variable thicknesses was set for an initial definition of a restricted pool of promising filters.

**Fig. 1:**
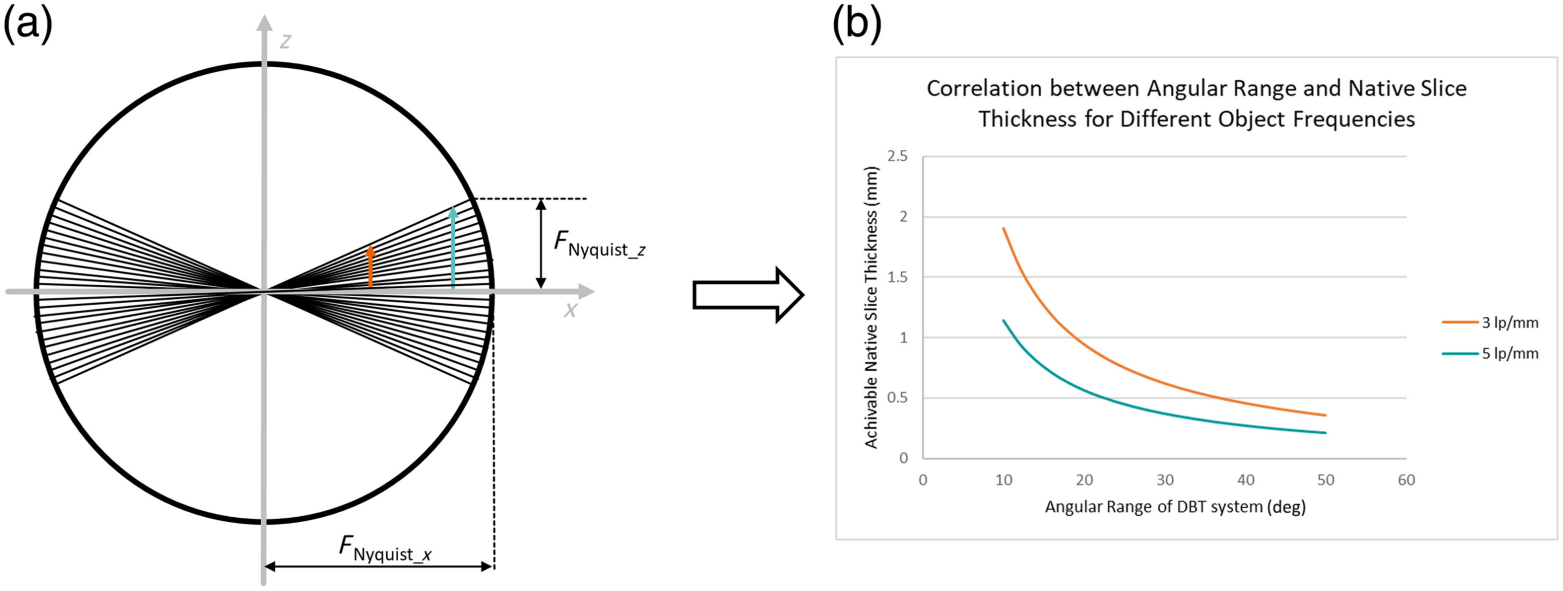
(a) Representation of the k-space filled with 25 projections over an angular scan range of 50 deg (black lines). The colored arrows illustrate the resulting depth information available for structures of two sizes, corresponding to 3 and 5 lp/mm. (b) Depth information under variation of the angular scan range. The larger the structures (the lower the frequencies), the more interference with adjacent structures occurs, and the narrower the angular scan range is chosen, the worse the interference becomes. Native slice thickness is defined as the reconstructed slice thickness where two adjacent slices may have different contents.

In FFDM, soft tissue contrast of the breast is mainly determined by the X-ray spectrum. In DBT, a second effect influences the final perceived contrast: this is the inherent suppression of anatomical background noise due to the tomographic effect. The number of projections in conjunction with the angle (θ) of DBT acquisition defines the data fill of the k-space (Fig. 1). The maximum frequency in the z-direction ($F_{\mathrm{Nyquist}\_z}$) defines the minimum native slice thickness meaning that two adjacent native slices may have different information contents. Native slice thickness d depends on the image frequency f and can be calculated according to the following equation:

$$d(f)=\;\frac{1}{2\cdot f\cdot \tan(\theta /2)}.$$

The parts in the k-space that do not contain information (white area) are linked to the out-of-plane artifacts in a DBT stack. Another interpretation is that the extent of frequency coverage in the z-direction goes along with the ability to separate overlying structures at this specific frequency in the x-direction. Large structures (mid- and low-frequency components) cannot be localized so well in the z-direction and thus cause more out-of-plane artifacts than small structures (high-frequency components). The larger the DBT angle, meaning the larger the fill of the k-space, the more depth separation, and thus, perceived contrast improvement is gained compared with FFDM. This gain in contrast plays a key role when fibroglandular structures are present in the breast that tend to consist of mid- and low-frequency components and thus create a large area of so-called anatomical noise which again can be reduced by a wider angular range.^4^ In addition, small, low-contrast structures such as spiculations or the outline of microcalcifications benefit from thin native slices which fully sample up to the Nyquist frequency defined by the scanning angle. In a post-processing step, native slices can be merged in a way that the contrast of these objects is preserved while presenting thicker slices to the clinician.

### 2.2 Tube Traveling Speed and Its Influence on Image Quality

**Fig. 2:**
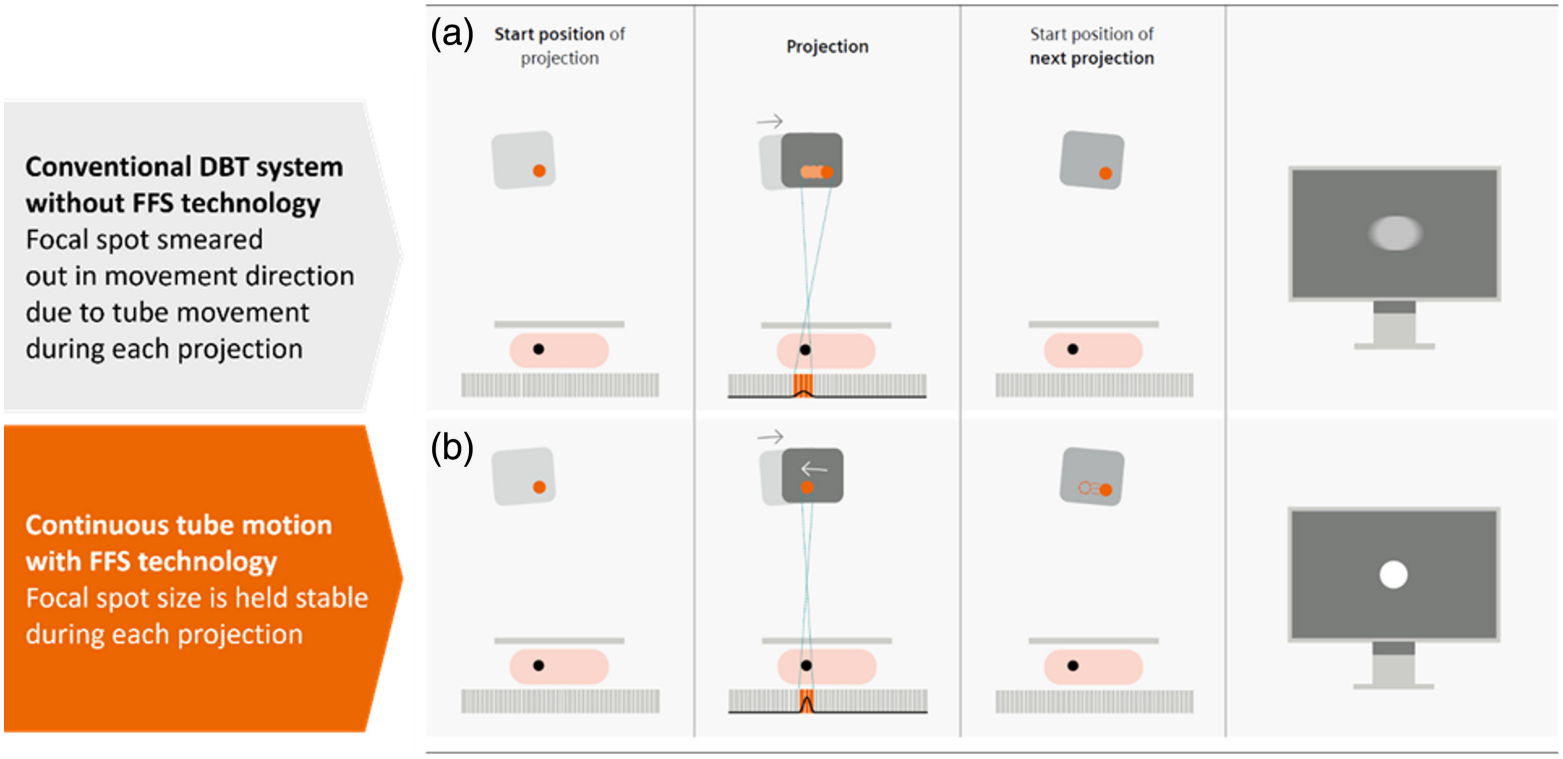
Schematic illustration of the tube and focal spot movement observed from the outside. (a) Top row illustrates a projection using continuous tube motion without FFS. The focal spot size is smeared out in the tube motion direction, resulting in a blurred visualization of the object. (b) Bottom row illustrates that projections using continuous tube motion with FFS, which compensates tube motion by deflecting the focal spot position during X-ray pulses into the opposite direction, results in a sharper visualization of the object.

**Fig. 3:**
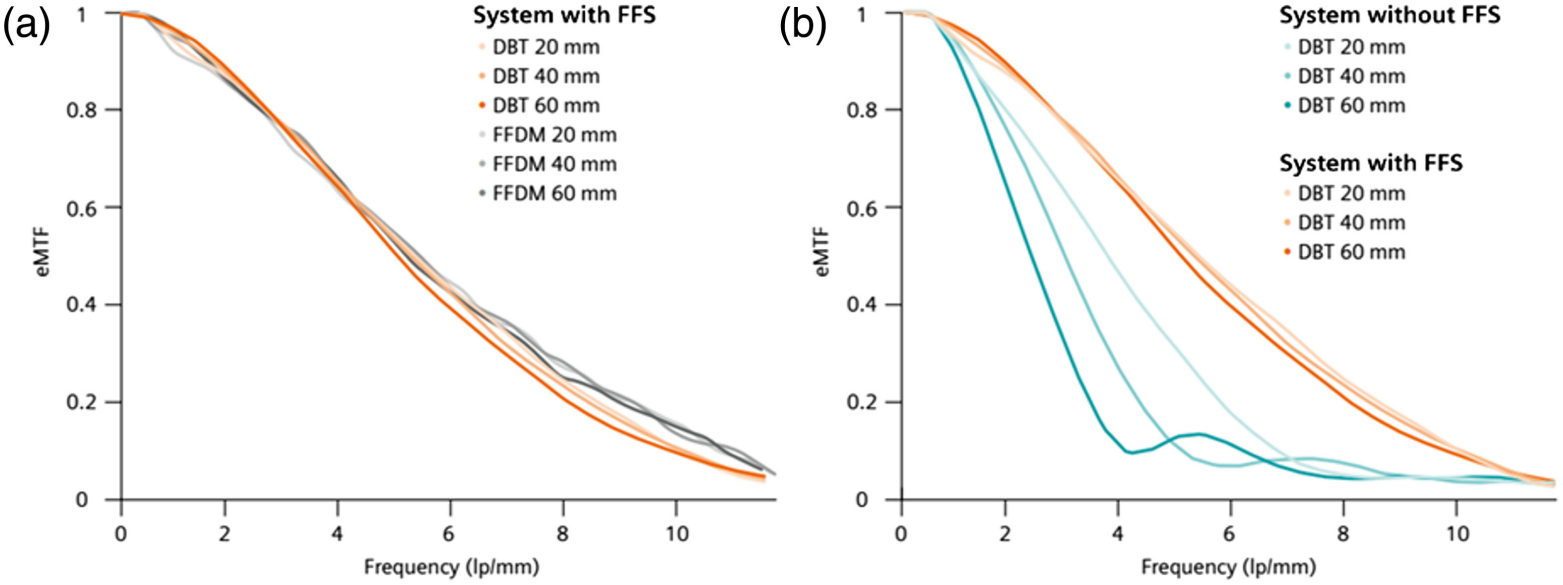
Effective MTF curves from the new system concept with an FFS (MAMMOMAT B.brilliant), averaged across all 25 projections, in the tube movement direction using a wire test object positioned at 20, 40, and 60 mm above the breast support table in comparison with (a) FFDM scans on the same system and (b) DBT scans acquired on a conventional wide-angle system without FFS technology (MAMMOMAT Revelation). The higher the plane of interest (larger magnification), the more the effect of the focal spot size (penumbra) influences the in-plane resolution.

For a given tube power, tube traveling speed does influence the in-plane resolution as the global focal spot movement during X-ray exposure enlarges the effective focal spot size per projection [see Fig. 2(a)]. The size of a focal spot plays basically no role for an area of interest directly on top of the detector but becomes more and more the leading factor for in-plane resolution when moving the slice of interest toward the tube. Effective modulation transfer functions (eMTFs) at different heights show this effect clearly [Fig. 3(b), petrol curves]. In addition, Fig. 3(b) demonstrates that results based on standard MTF measured at the detector height are not representative of the clinical practice where the calcifications are located between 2 and 7 cm in height. Although image frequencies in the range of 4 lp/mm can be visualized at a height of 20 mm, these vanish at a height of 60 mm. The optimization scheme for a conventional system should therefore address patient movement risk and patient discomfort as well as preserve as much as possible in-plane resolution at clinically relevant heights.

### 2.3 Reconstruction Algorithms

To transfer the hardware improvements into better diagnostic images for the radiologist, the reconstruction framework is adapted accordingly. In addition, features that became possible by higher computational performance and recent developments, e.g., artificial intelligence, were added. Image perception and microcalcification perceptibility are a higher focus for SM and the DBT stack.

#### 2.3.1 Image impression

Image impression, often referred to as “flavoring,” can be adjusted to better integrate into different clinical setups and to meet radiologists’ preferences more effectively. This is achieved by customizing sharpness, contrast, and brightness, as well as by suppressing dark overshoot artifacts through the adjustment of specific image frequency bands. These enhancements allow for contrast and brightness settings beyond normal window/level adjustments.

The adaptation of image impression is realized through a two-step approach. The first step addresses widespread gray-level variations due to the differences in breast thickness or density, particularly at the breast border versus the fully compressed region. As tomosynthesis is not full tomography, these thickness variations still influence the reconstructed slices. This step establishes a baseline for the second step, which involves multifrequency processing of the DBT slices, allowing individual frequency bands to be processed independently.

This method ensures that the image impression can be tailored to specific clinical environments, enhancing overall diagnostic effectiveness.

#### 2.3.2 Advanced artifact reduction

Larger highly attenuating objects such as biopsy markers, surgical clips, wires, macrocalcifications, and needles produce two distinct kinds of artifacts in DBT reconstructions. First, in-plane artifacts result in dark shadows around the object in slices where the object is in focus. Second, out-of-plane artifacts result in bright traces of the object in adjacent slices where the object is out-of-focus. The high attenuating object overlaps the tissue inside the shadow for some of the acquired projections, which leads to areas with inconsistent object and non-object pixels in the different projection angles.

A method was designed to reduce these artifacts.^21^^,^^22^ First, a segmentation in the projection domain is performed to identify objects with a larger contrast difference to the surrounding tissue. The segmentation is based on a background estimation with morphological operations, and the selection of the structural elements ensures that objects above a certain size, e.g., implants, will not be segmented due to potential over-correction. Very small high-contrast elements are also removed from the segmentation as they do not create these artifacts. For the backprojection, two sets of images are prepared, the ramp-filtered original projections (oP) and projections with virtually removed high-contrast objects (rP). In the backprojection part of the reconstruction process, the segmentation masks will be used to select the areas without information (artifacts) so that for the affected voxels, the rP will be used to recover the missing information. To avoid side effects, segmentation and artifact area selection are done in a conservative way; thus, traces of artifacts on low-contrast objects or objects with frayed borders might still be present in the images. Regions in the shadow of, for example, large macrocalcifications still show traces of artifacts because only a very small part of the acquired projection images will contain usable data due to the shielding effect of the object.

#### 2.3.3 Perspective coordinate system

**Fig. 4:**
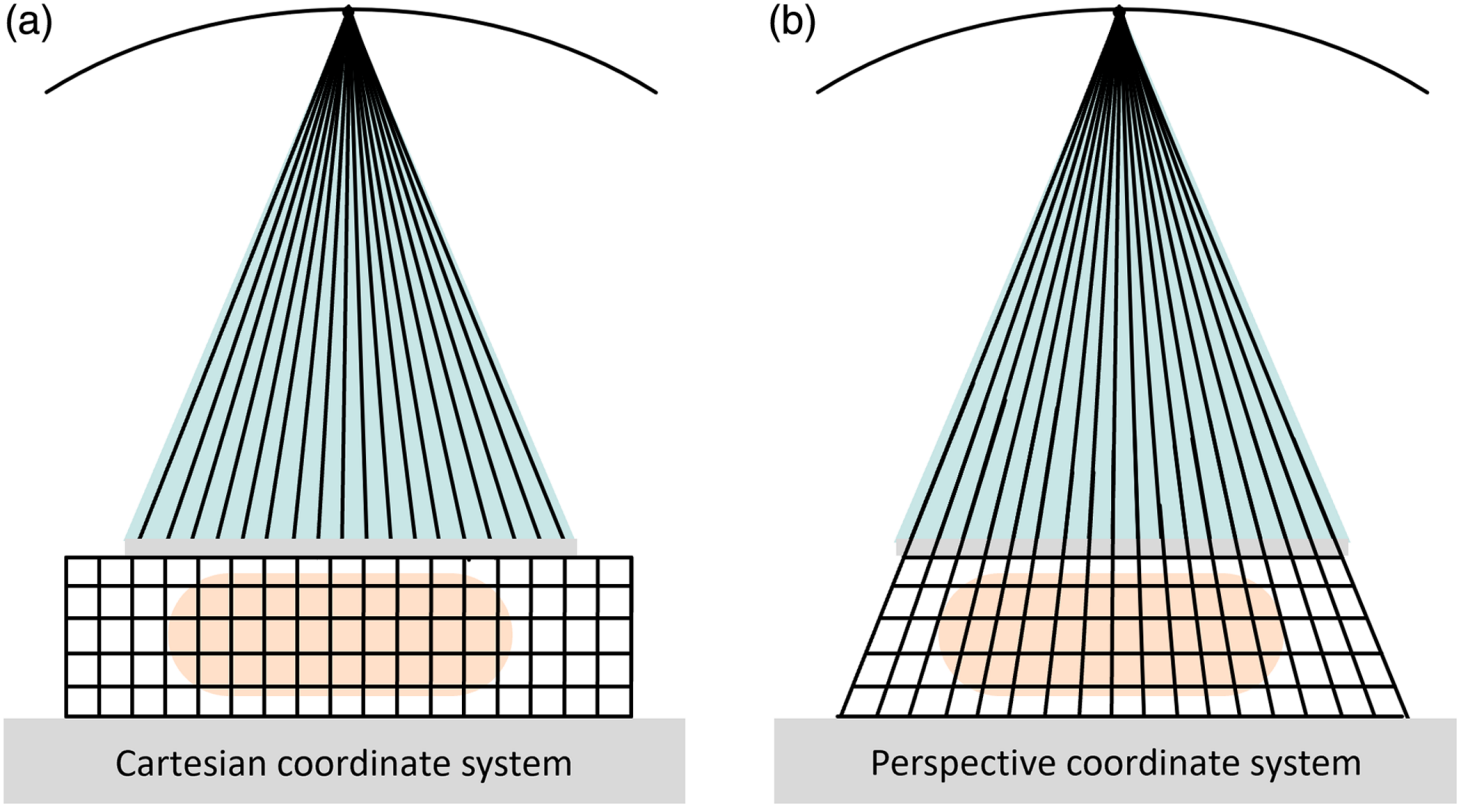
In contrast to the fixed voxel sampling distances and sizes in all slices in the Cartesian coordinate system (a), sampling distances and sizes of voxels are adapted to the central view position (0 deg) in the perspective coordinate system (b).

To exploit the higher spatial resolution of the raw projection images in DBTs and SMs, the concept of a perspective coordinate system is used. In contrast to a Cartesian coordinate system, the sampling of volume voxels is performed along the rays of the central projection instead of using overall fixed positions in all slices (see Fig. 4). Rays along the central projection all have the same in-plane index positions and can be combined without a perspective forward projection which would introduce an additional interpolation step. This allows for both optimal image resolution and reduced computational effort at the same time. All internal calculations are carried out in this coordinate system as this is the native cone beam geometry representation. As one of the last steps in the DBT reconstruction pipeline, there is the possibility to change the data representation from the perspective coordinate system to a Cartesian one, if desired.

Besides avoiding an interpolation step as, for example, required for the generation of the SM, the use of the perspective coordinate system also offers multiple advantages regarding the image impression: When scrolling through the slices, the point-spread response of microcalcifications will remain static regarding their in-plane location. The same is true for background noise, structures, or remaining traces of artifacts by high-contrast objects that could not be fully compensated by artifact reduction. When using slabbing technology at a viewing station, structures will keep their sharpness and structure when multiple slices are averaged.

#### 2.3.4 Physics-constrained artificial intelligence (AI) noise reduction

Recent developments in machine learning offer novel approaches to problems that so far have been tackled with classical analytical methods. The denoising of images is such an example, which in the past was approached via classical filter algorithms such as Gaussian smoothing, mean or median filtration, or using more sophisticated iterative approaches that try to compute noise probabilities based on local neighborhood or prior information. The most prominent problem with all those methods is the tendency to either get smeared results or an artificial-looking image impression. The generation of SMs is usually based on background information from a limited amount of DBT projection images enriched with information from the DBT stack such as calcifications or prominent structures. DBT projection images carry a relatively low dose, and the noise level needs to be adjusted for optimal synthesis with DBT slice image data, which carries important detailed information such as microcalcifications. It was found that AI-based denoising of DBT projection images is well suited for this task. The method yields noise estimates of each projection and is designed to be bound to the physics laws of noise.^23^ In addition, a plausibility filter is used to constrain the AI model’s noise estimate before subtraction from the image data. This avoids the removal of statistically relevant information and prevents pseudo-structures from arising.^24^ Kindly note that fine structures that are below the noise limit will, such as with any other denoising method, not be recovered.

## 3 Results and Discussion

### 3.1 Optimization of the Spectrum and Its Dependency on the DBT Angle

As the acquisition techniques of FFDM and DBT differ significantly, spectral optimization was performed separately. However, a high similarity of the resulting image contrast was a purposefully chosen design constraint, to not increase complexity for the radiologists when looking at FFDM and DBT side-by-side. A multi-parameter optimization scheme was used in combination with expert decision rating drawbacks versus benefits. The simulation was conducted with fixed components such as an a-Se detector with a pixel pitch of 85  μm, an X-ray generation unit of 5 kW peak power, and a tungsten anode. Optimization parameters were patient experience, patient movement artifacts, image contrast at a specific dose (${\mathrm{CNR}}^{2}/\mathrm{AGD}$), signal-to-noise ratio at the detector, electrical energy consumption of the tube, breast thickness distribution, and current imaging bottlenecks. For DBT, a filter material with no K-absorption edge in the relevant energy range was chosen as it offers pronounced advantages especially for dense and/or thicker breasts: electrical tube power is used more effectively, which enables fast scanning and reduces the risk of patient movement in DBT.

**Fig. 5:**
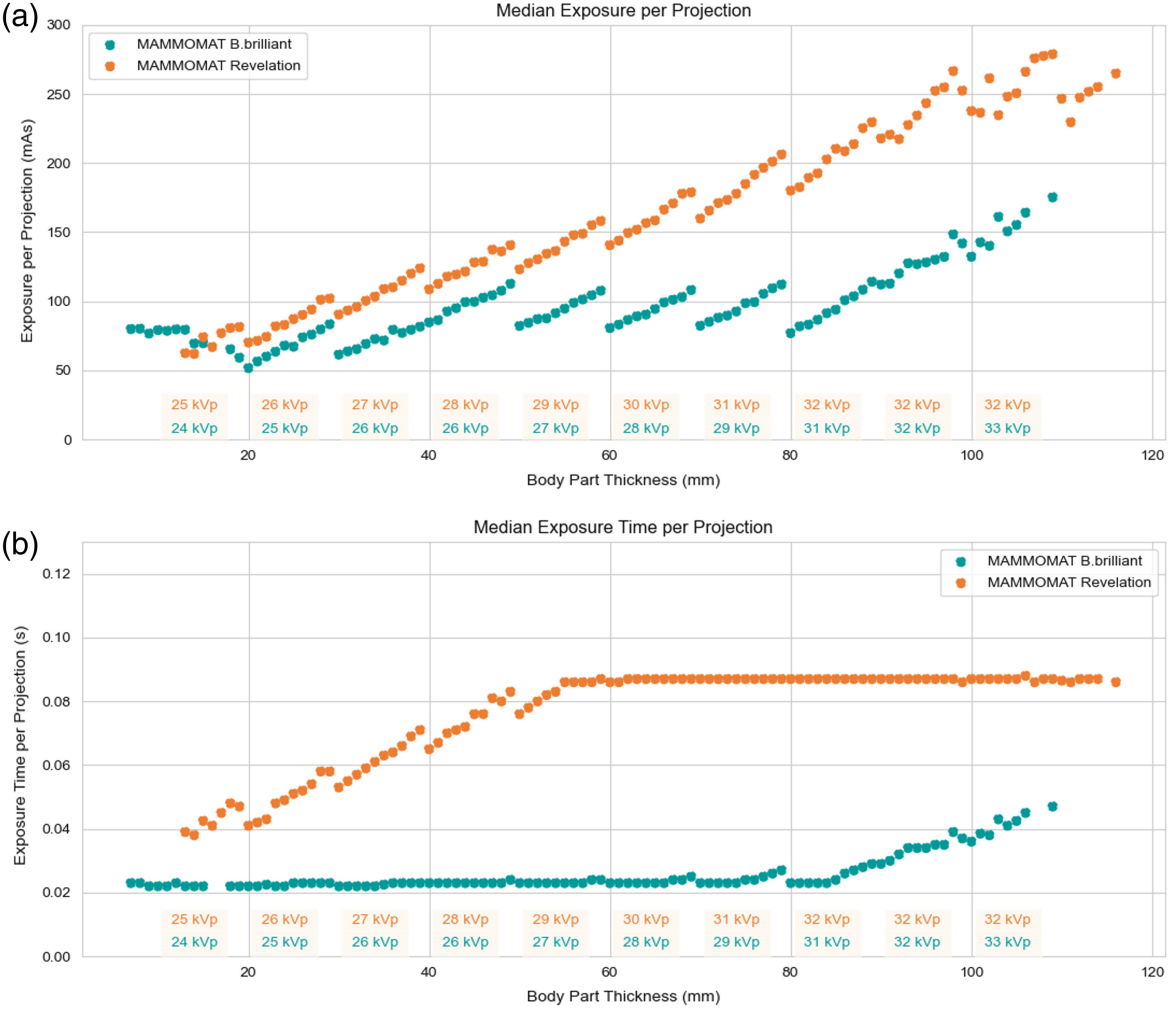
Log data analysis of MAMMOMAT Revelation (orange dots) and the novel system concept MAMMOMAT B.brilliant (petrol dots). The corresponding kVp values are linked to the body part thickness and can be found at the bottom of the figures. They were chosen to reach the highest ${\mathrm{CNR}}^{2}/\mathrm{AGD}$. (a) Median exposure values. The kVp steps can be seen clearly in the data as a saw tooth pattern. For a body part thickness above 50 mm, a clear advantage can be found for the new filtration. (b) Overall advantage in terms of a significantly reduced exposure time per projection for the new system filtration and system control.

As a result, an aluminum filtration with a thickness of 0.7 mm was selected. The resulting effects in terms of exposure characteristics can be seen in the data from a clinical DBT evaluation in Fig. 5. Compared to 0.05-mm rhodium filtration used in established systems, we observe a clear reduction in tube power and exposure time.

To maintain the desired high similarity in image contrast, aluminum filtration was also selected for FFDM. A 1.0-mm thickness configuration was chosen, as it yields slightly better ${\mathrm{CNR}}^{2}/\mathrm{AGD}$ values whereas tube power and exposure time are not as critical in FFDM.

### 3.2 Tube Traveling Speed and Its Influence on Image Quality

For the novel system concept, an X-ray tube with an adaptive focal spot position based on the flying focal spot (FFS) technology was used. With an electromagnetic coil inside the single tank unit, one can influence the electrons on their way from the emitter to the anode. By applying ramped coil currents, the focal spot can be moved alongside the beam track of the anode. It is controlled in a way so that during the X-ray projections, the position of the focal spot during tube motion is held effectively stable with respect to the breast and detector, as illustrated in Fig. 2(b). As the traveling length of the electrons on their way from the emitter to the anode increases with larger deviations of their middle position, the emitter component was designed in a way that the focal distance is larger than the distance between the emitter and the anode. This means that the focal spot size marginally shrinks when being deflected. The flying focal spot technology eliminates blurring effects, and the effective focal spot size is no longer impaired by the tube motion speed. With this approach, much faster DBT scans with concurrently higher in-plane resolution can be achieved.^25^ Measurements of the eMTF were performed to analyze the effectiveness of the FFS technology. Figure 3 compares the height-dependent in-plane resolution of DBT with FFDM and compares it to a conventional DBT system without flying focal spot technology. The eMTF results for MAMMOMAT Revelation are in accordance with a previous investigation from Marshall et al.^25^ Marshall et al. compared eMTF curves from multiple DBT systems, which showed all inferior results in the tube movement direction compared with those achieved in this study with the new system concept of the MAMMOMAT B.brilliant. However, this has to be confirmed in further research under similar test conditions. Based on our measurements, it is clearly visible that the new system concept is on the same level as FFDM and does outperform MAMMOMAT Revelation. The effective focal spot size (penumbra) has a stronger negative impact on the achievable in-plane resolution at larger distances from the detector (larger magnification). The in-plane resolution of the MAMMOMAT Revelation is limited by the effective focal spot size, whereas the effective focal spot size of the MAMMOMAT B.brilliant is small enough at 60 mm so that the in-plane resolution is still dominated by the pixel pitch of the detector.

Incorporating a breast-specific acquisition speed was found to be necessary; therefore, two scanning modes were introduced to cover the variability of tube energy requirements (Table 1). The system control chooses automatically which mode will be used based on the exposure parameters (kVp and mAs). Effective MTF evaluation does not show any difference in either mode.

**Table 1:** Comparison of acquisition modes.

	Fast mode	Moderate fast mode
Acquisition time	4.8 s	8.1 s
Frames per seconds	5	3
Number of projections	25	25
Angular range	50 deg	50 deg
Max X-ray pulse length	40 ms	68 ms
Read-out time of detector	150 ms	150 ms
Tube traveling speed	10.4 deg/s	6.0 deg/s
Prevalence in clinical practice (screening and diagnosis)^a^	98%	2%

a Based on 95,329 scans acquired with 43 MAMMOMAT B.brilliant systems in the field.

### 3.3 New Reconstruction Algorithm

**Fig. 6:**
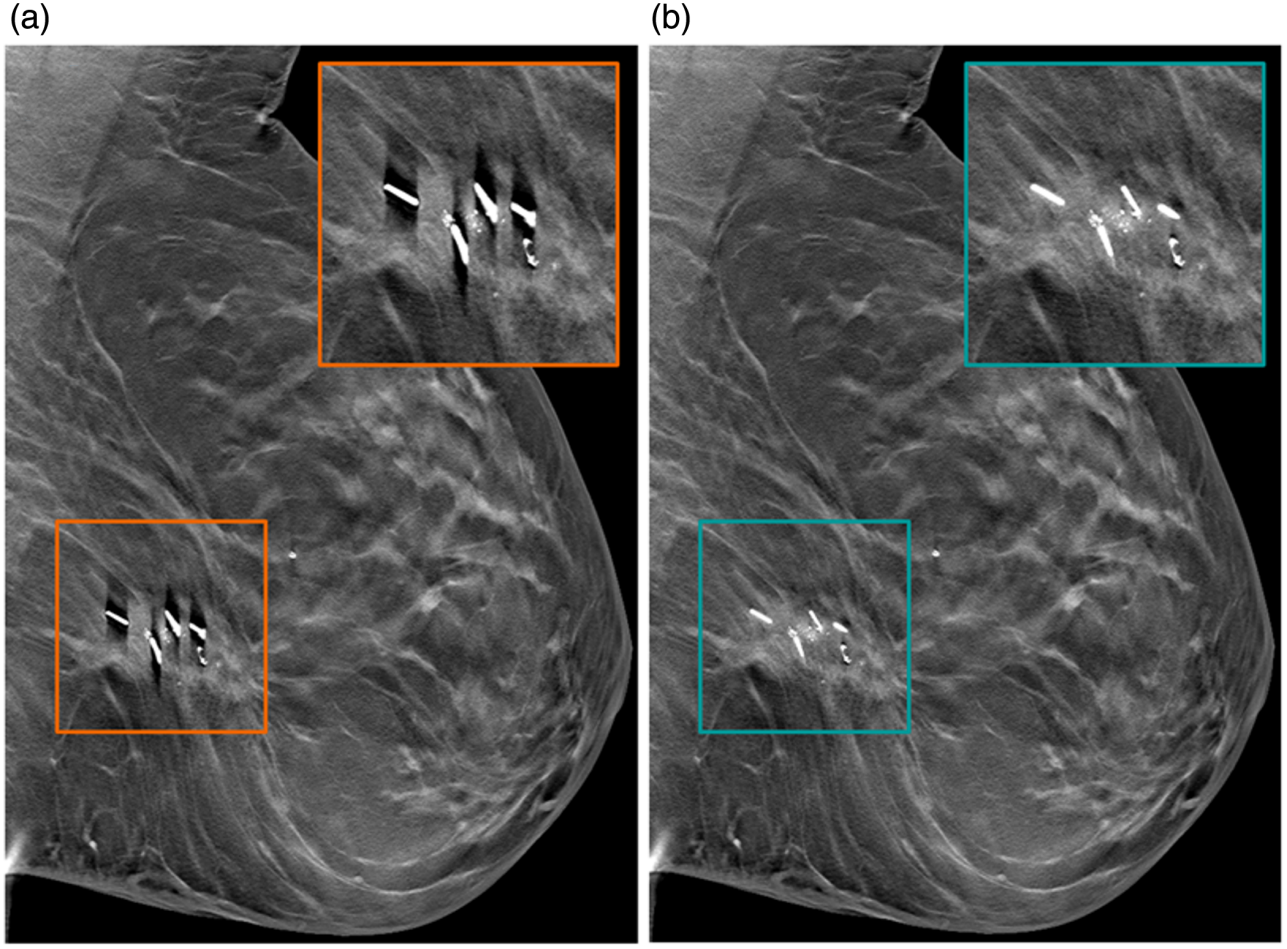
Comparison of a left mediolateral oblique view without (a) and with the new artifact reduction method (b). Strong overshoots caused by high-contrast edges are clearly suppressed and allow for better visibility of the surrounding tissue.

**Fig. 7:**
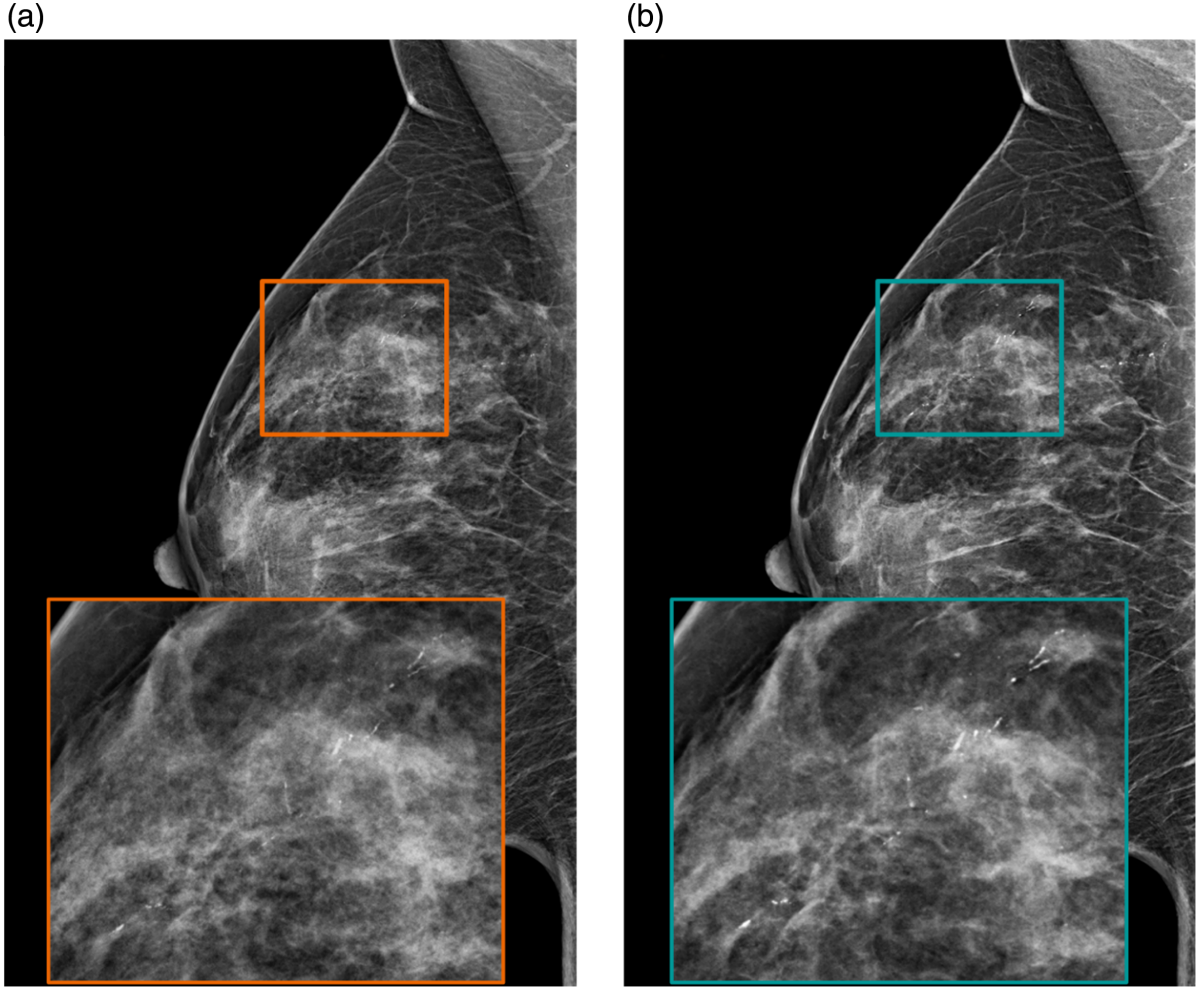
Clinical case with microcalcifications seen on FFDM (a) and in the SM computed from DBT (b) corresponding to ductal carcinoma *in situ* grade 3 lesion.

For an illustration of the resulting images after applying the new reconstruction with the new DBT system, Fig. 6 shows a mediolateral oblique DBT view from a patient with multiple clips in her left breast after surgery. The DBT image was reconstructed without and with the advanced artifact reduction algorithm to demonstrate its effect. Figure 7 shows microcalcifications seen in the right mediolateral oblique view of both FFDM and SM acquired under the same compression. Both patients gave informed consent that their data could be further used and processed for publication purposes. Reconstruction was set to a slice thickness of 2 mm and a slice distance of 1 mm.

## 4 Conclusion

The newly introduced system concept expands the benefits of wide-angle DBT by fast scanning and high in-plane resolution. The stationary focus position, resulting from the FFS technology, leads to a significantly improved eMTF in wide-angle DBT, with FFDM-like in-plane resolution over a broad range of breast thicknesses. Laboratory tests and clinical cases demonstrate clearly that the gain in sharpness can be exploited in both DBT and SM images. This results in improved small-detail detectability, especially for objects located at clinically relevant heights above the detector. Furthermore, the shorter scan time reduces the likelihood of patient motion artifacts, which is another crucial factor for improving microcalcification visibility. The presented technology shows strong potential to be a key contributor to facilitating the paradigm shift of replacing FFDM with DBT plus SM in breast cancer screening.

## Data Availability

The data cannot be made publicly available upon publication due to legal restrictions preventing unrestricted public distribution. The data supporting this study’s findings are available upon reasonable request from the authors.
